# To migrate, stay put, or wander? Varied movement strategies in bald eagles (*Haliaeetus leucocephalus*)

**DOI:** 10.1186/s40462-017-0102-4

**Published:** 2017-05-05

**Authors:** Rachel E. Wheat, Stephen B. Lewis, Yiwei Wang, Taal Levi, Christopher C. Wilmers

**Affiliations:** 1 Center for Integrated Spatial Research, Department of Environmental Studies, University of California, Santa Cruz, 1156 High Street, Santa Cruz, CA 95064 USA; 2 U.S. Fish and Wildlife Service, 3000 Vintage Blvd., Suite 201, Juneau, AK 99801 USA; 3 San Francisco Bay Bird Observatory, 524 Valley Way, Milpitas, CA 95035 USA; 4 Department of Fisheries and Wildlife, Oregon State University, 104 Nash Hall, 2820 SW Campus Way, Corvallis, OR 97331 USA; 5 Center for Integrated Spatial Research, Department of Environmental Studies, University of California, Santa Cruz, 1156 High Street, Santa Cruz, CA 95064 USA

**Keywords:** Movement strategies, *Haliaeetus leucocephalus*, Space use, GPS, Brownian bridge movement modeling

## Abstract

**Background:**

Quantifying individual variability in movement behavior is critical to understanding population-level patterns in animals. Here, we explore intraspecific variation in movement strategies of bald eagles (*Haliaeetus leucocephalus*) in the north Pacific, where there is high spatiotemporal resource variability. We tracked 28 bald eagles (five immature, 23 adult) using GPS transmitters between May 2010 and January 2016.

**Results:**

We found evidence of four movement strategies among bald eagles in southeastern Alaska and western Canada: breeding individuals that were largely sedentary and remained near nest sites year-round, non-breeding migratory individuals that made regular seasonal travel between northern summer and southern winter ranges, non-breeding localized individuals that displayed fidelity to foraging sites, and non-breeding nomadic individuals with irregular movement. On average, males traveled farther per day than females. Most nomadic individuals were immature, and all residential individuals (i.e. breeders and localized birds) were adults.

**Conclusions:**

Alternative movement strategies among north Pacific eagles are likely associated with the age and sex class, as well as breeding status, of an individual. Intraspecific variation in movement strategies within the population results in different space use patterns among contingents, which has important implications for conservation and management.

**Electronic supplementary material:**

The online version of this article (doi:10.1186/s40462-017-0102-4) contains supplementary material, which is available to authorized users.

## Background

Movement strategies are widely acknowledged to be a critical component of the survival and fitness of most organisms [1, 2], as food and other important resources can be distributed heterogeneously across both time and space. Theory predicts that animals should minimize the effects of this heterogeneity in resource availability by moving among areas of high resource abundance and limiting time in areas with low resource abundance [3–5]. Quantifying individual variability in movement behavior is critical to understanding population-level patterns in animals. Identifying individual movement strategies can help researchers understand the flexibility with which animals can respond to variable environmental conditions and design more effective conservation management plans [6, 7].

Depending on the extent of resource variability, some movement strategies might prove more effective for resource acquisition than others, and we expect natural selection to favor movement strategies that maximize resource acquisition [8]. Resources with little variation in spatial heterogeneity, for example, should facilitate a sedentary strategy, whereas resources with predictable seasonal variation should generate migratory movement strategies within populations. If resource heterogeneity is extensive both spatially and temporally (i.e., spatially variable and temporally unpredictable), nomadism should emerge as the favored movement strategy [9, 10].

While much empirical evidence exists regarding variability of movement strategies among different species, evidence is somewhat less prevalent for the extent of variability of movement strategies within a species [7, 10]. Individuals in many species can adapt to varied environments and adjust behavioral strategies to facilitate resource acquisition through time, by, for example, bypassing low-quality sites or engaging in exploratory behavior to locate new resource patches [11]. Thus, in some environments it is likely that selection for optimal strategies and behavioral flexibility in individuals is strong.

Intraspecific variation in movement strategies may be particularly prevalent in environments in which resources are highly variable, as individuals that can learn when and where to access the best resources and alter their movement strategies accordingly will be those most likely to survive and reproduce [12]. Partial migration, where populations are composed of a mixture of migratory and residential individuals, is widespread, with examples across all major vertebrate groups [13], but few studies have explored variation in movement patterns beyond this dichotomy. One study, by Singh et al. [7], found evidence of four different movement strategies within moose (*Alces alces*) across seven latitudes in Sweden: migratory, disperser, nomadic, and resident. Similarly, Austin et al. [8] found that grey seals (*Halichoerus grypus*) near Nova Scotia, Canada could be separated into three different movement groups: residents, seals that moved in a random fashion, and seals that undertook long distance, directed movements. Here, we explore intraspecific variation in the movement strategies of a highly mobile species in a large-scale, highly heterogeneous environment, using bald eagles (*Haliaeetus leucocephalus*) along the north Pacific coast as a case study.

While food resources for almost all animals are variable across time and space, the north Pacific coast presents an extreme case in which anadromous fish and other prey are widely dispersed across the landscape in discrete patches that are asynchronous, but temporally abundant and generally predictable [14]. The north Pacific coast of North America provides spawning habitat for more than 15 species of anadromous fishes. Seven species of Pacific salmon and trout (*Oncorhynchus*; Salmonidae), at least one species of smelt (Osmeridae), three species of lamprey (Petromyzontidae), and at least one species of char (*Salvelinus*) spawn in fresh water on the coasts of northern California, Oregon, Washington, British Columbia, and Alaska [14–16], in addition to several species of non-anadromous forage fish such as Pacific herring (*Clupea pallasii*). The timing and locations of adult in-migration, spawning aggregations, and smolt out-migration of anadromous fish, and the spatiotemporal availability of other fish, varies extensively among species and among populations in different locations but are generally predictable year to year [16, 17].

Bald eagles are highly mobile consumers prevalent throughout the northwestern United States and western Canada. Although they are considered generalist predators [18], in coastal regions upwards of 90% of eagle diets are comprised of fish [19]. Bald eagles display delayed maturation, in that most individuals do not reach sexual maturity until their fifth or sixth calendar year [18], and it may take even longer to earn a breeding territory in the likely-saturated breeding population of the north Pacific [20]. Bald eagles are thought to be partially migratory [17]; historical research on eagle movements along the north Pacific coast suggests that adults may be residential while immature birds move south in late autumn as abundant food resources (e.g., salmon runs) are exhausted [20, 21]. Counts of eagles along the British Columbia, Canada coast suggest an influx of eagles (presumably from coastal southeastern Alaska) during winter [22].

Despite considerable interest in the species, little is understood about the extent of intraspecific variation in movement strategies among bald eagles. While movement patterns of bald eagles are generally well-known, technological advances in the capabilities of tracking devices now permit exploration of animal movement on fine spatial and extended temporal scales [23]. Understanding the extent of intraspecific variation in eagle movements could have important implications for conservation and management. Management plans that fail to account for multiple movement strategies might be limited in their effectiveness. For example, management strategies focusing solely on migratory animals may result in localized depletion of residential animals [24]. Additionally, maintaining contingents that utilize differing movement strategies within a population may be important in mediating population stability [25].

Our objective here was to use fine-scale GPS tracking data to examine variability in intraspecific movement strategies among individual eagles. We used Brownian bridge movement modeling to examine home ranges of individual eagles and to delineate areas of high intensity use along coastal areas in southeastern Alaska and western Canada for each movement strategy. We predicted that (i) given the extreme heterogeneity of resource availability across the region, behavioral flexibility among eagles would be strong, resulting in multiple movement strategies, (ii) at the population level, migratory movement strategies should be favored, given the predictable nature of anadromous and other fish runs, and (iii) areas of highest-intensity use would vary seasonally and among different movement strategies.

## Methods

### Study area

Our study area encompassed the north Pacific coast and surrounding region from Vancouver Island, British Columbia north to the top of southeastern Alaska, northwest of Yakutat Bay. The area is characterized by steep, rugged topography, numerous islands, coastal fjords, and large tracts of temperate rainforest. The landscape is naturally fragmented by mountainous terrain, wetlands, and various fine-scale disturbances (e.g., wind-throw) as well as varying degrees of anthropogenic disturbance (e.g., clear-cut logging). The forests are dominated by western hemlock (*Tsuga heterophylla*) and Sitka spruce (*Picea sitchensis*) in the north and western hemlock, Douglas fir (*Pseudotsuga menziesii*), and western red cedar (*Thuja plicata*) in the south. A cool and wet maritime climate characterizes the region.

### Captures and tracking

We captured bald eagles in four locations in southeastern Alaska: in Icy Bay (59°57′55.80″N, 141°25′49.71″W; *N* = 9) in May 2010, 2011, and 2012; in Juneau (58°21′13.65″N, 134°36′12.04″W; *N* = 2) in March 2011 and June 2014; in Sitka (57°02′36.58″N, 135°22′50.19″W; *N* = 5) in March 2011; and on the Chilkat River (59°14′58.20″N, 135°35′25.19″W; *N* = 12) in November 2012 and 2013. In Icy Bay, Juneau, and Sitka we captured eagles on near-shore waters of the ocean using a modified floating-fish snare [26, 27]. We trapped bald eagles on gravel bars along the Chilkat River using perch snares [20] or a radio-controlled bow net (Superior Bownet & Design, Washington, MD, USA) or rocket net (Coda Enterprises, Mesa, AZ, USA) baited with salmon carcasses. Eagles were outfitted with 70 g solar-powered GPS/PTTs (Microwave Telemetry, Inc., Columbia, MD, USA) using a backpack-style Teflon ribbon harness. All birds were banded and aged based on plumage [28, 29]. A blood sample was taken, and sex was determined using DNA from sex chromosomes (Zoogen, Inc., Davis, CA, USA). Eagle capture and handling methods were in compliance with Institutional Animal Care and Use Committee protocols at the University of California, Santa Cruz (Wilmc1206) and the U.S. Fish and Wildlife Service (2010004).

We tracked 28 individual eagles between May 2010 and January 2016. Tracking periods ranged from 6.8 months to 4.8 years, and averaged 2.5 years ± 2.8 months (Additional file 1). GPS units were configured with duty cycles that shifted based on time of year to conserve battery life during shorter days. Units were programmed to gather hourly GPS locations 1 October through 28 February from 08:00 to 17:00, 1 March through 30 April and 1 August through 30 September from 05:00 to 19:00, and 1 May through 31 July from 03:00 to 22:00 AKT. Each GPS unit transmitted to the Argos satellite system (CLS America, Lanham, MD, USA) for four hours every other day during spring and summer and every fourth day during autumn and winter. We parsed data using the manufacturer’s software. Units yield locations with a horizontal accuracy of up to ± 18 m (Microwave Telemetry, Inc.). As a result of low battery voltage, likely related to insufficient insolation, signals from some transmitters were intermittent, lapsing for days or occasionally weeks at a time, particularly mid-winter, when daylight is limited. In our analyses, we include only data unaffected by such lapses.

### Movement strategies

**Fig. 1:**
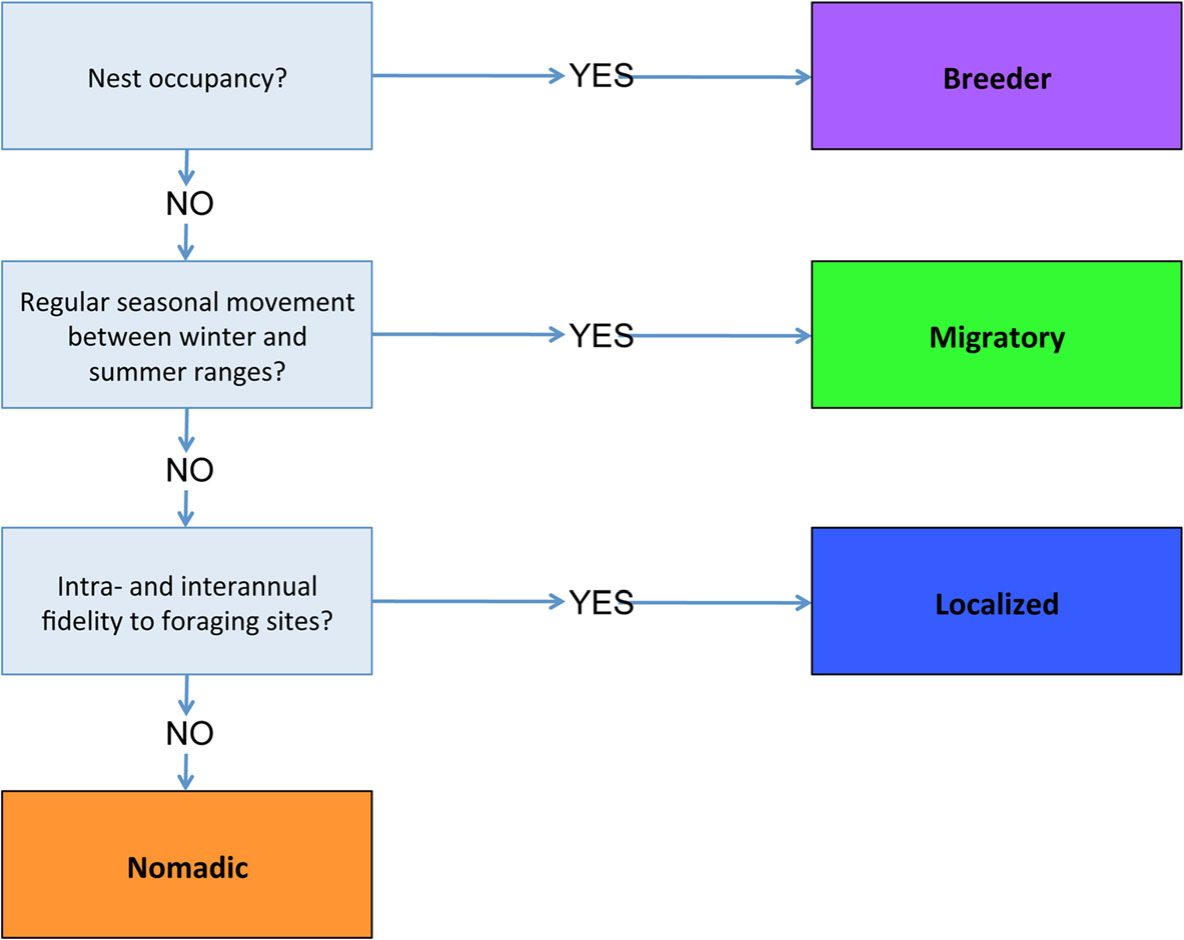
Movement strategy classification flow chart

We plotted data in ArcGIS 10 (ESRI, Redlands, CA, USA) for visual inspection, removal of erroneous data, and data analysis. We recognized four types of movement strategies and grouped individual eagles into one of four contingents according the flow chart in Fig. 1, based on observed movement patterns: breeder, localized non-breeder, non-breeding migratory, or non-breeding nomadic. During the breeding season (April and May), we flagged potential breeders by identifying clusters of relocations at a single site. Clusters of relocations occurring within a 500 m radius across four or more days were investigated for potential nesting behavior. Visual confirmation of nesting behavior (e.g. presence of the individual on or near a nest) via fixed-wing aircraft or boat resulted in classification as breeder, regardless of whether any breeding attempt was successful. We defined regular seasonal movements, used in classifying migratory individuals, as movements that follow the same general route and timing from year to year, with seasonal ranges defined by movements restricted within a 150 km^2^ area. Site fidelity, used in classifying localized versus nomadic individuals, was confirmed if an individual returned after more than three months to within 50 km^2^ of a previously-visited site.

We compared movement strategies, as well as sex and age classes, using average distance traveled per day and utilization distributions. For each individual, we calculated step length as the straight-line distance between consecutive GPS fixes. To reduce bias towards larger step lengths that may result from lapses in signal and travel occurring between duty cycles, we removed all distance values from fixes that were greater than 15 h apart, the maximum possible gap between duty cycles. We then calculated average daily distance traveled by month for each individual. We calculated home range sizes for each individual from utilization distributions (UDs) generated using Brownian bridge movement modeling (BBMM; [30]) with the ‘move’ package in R [31]. We investigated potential differences in UDs and average daily distances traveled among movement classes, between males and females, and between adult and immature eagles using Kruskal-Wallis tests, and report means ± SE.

### Space use

Utilization distributions allow for the mapping of the intensity of use within a defined area [32]. Following Watts et al. [33, 34] we used Brownian bridge movement modeling to develop utilization distributions for each individual eagle using locations (*n* = 245,865) collected between May 2010 and January 2016. We used the R package ‘move’ [31] to produce UD surfaces using the dynamic Brownian bridge movement model (dBBMM) [35]. Window size of 21 was based on the maximum number of GPS locations received per day for an individual eagle. The margin was set in proportion to window size. Location error was determined by the transmitter manufacturer as ±18 m. In order to generate the most detailed output for the geographic scale of our study area, we set the cell size to 5 km^2^. We exported UDs as rasters and overlaid them across the study area in ArcMap.

We produced independent surfaces for all eagles individually for winter (January through March), spring (April through June), summer (July through September), and autumn (October through December) to reflect seasonal variation in movement. We combined UD surface maps produced for individual eagles by averaging probabilities for each cell to produce UDs for each movement strategy (breeder, localized, migratory, and nomadic) for each season. The number of locations in each movement track varied among individual eagles, so we weighted, combined, and standardized UD surfaces according to the number of locations per track.

For display purposes, we ordinated cell values from highest to lowest and grouped cells within categories that represented 10% of the total eagle utilization, such that the first category was comprised of the cells with the highest utilization. The first category represents the minimum area to achieve 10% of the total utilization, the second category reflects the minimum area to achieve the next 10% of the total utilization, etc. This presentation thus allows for visualization of the relationship between the intensity of use and area [34].

## Results

### Movement strategies

**Fig. 2:**
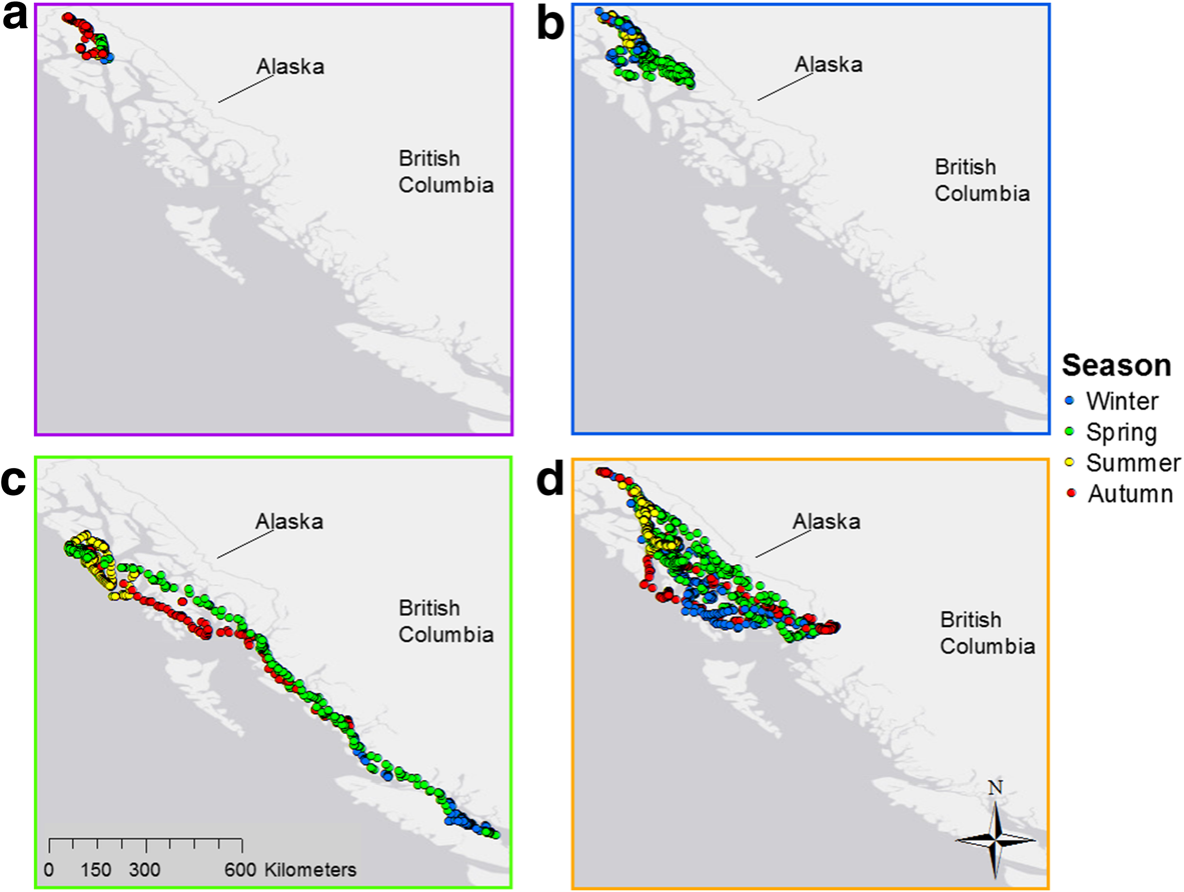
Examples of the four different strategies of movement observed in bald eagles monitored along the north Pacific coast, 2010–1016. Breeders (**a**) remained near nesting sites year-round, with short distance movements for access to seasonal resources (i.e. autumn). Non-breeding localized individuals (**b**) engaged in primarily short-distance movements for access to seasonal resources (i.e. spring, autumn) within a distinct range. Non-breeding migratory individuals (**c**) had directed moves in autumn and spring between distinct summer and winter ranges. Non-breeding nomadic individuals (**d**) displayed irregular movement patterns with little interannual consistency

Eagles classed as breeders were largely residential, remaining near nest sites year-round, with occasional short forays to nearby watersheds for access to seasonal resources (Fig. 2a). Individuals classed as migratory had no nesting territories and showed no evidence of breeding—these individuals displayed no fidelity to a nesting site, as indicated by a lack of clustering in a single location during the breeding season. Non-breeding migratory eagles displayed regular seasonal movement, following the same general route and timing from year to year, between separate winter and summer ranges, moving southward in autumn to a winter range and northward in spring to a summer range (Fig. 2c). Migratory individuals displayed interannual fidelity to winter and summer ranges. Eagles classed as non-breeding localized individuals had no fidelity to a nesting site and showed no evidence of breeding, but, unlike migratory individuals, made no regular seasonal movements between separate winter and summer ranges. Much like breeders, localized eagles were largely residential, with intra- and interannual fidelity to foraging areas. Localized individuals made seasonal forays to nearby watersheds for access to seasonal resources, ranging more widely than breeding birds (Fig. 2b). Individuals classed as nomadic had no fidelity to nesting sites and showed no evidence of breeding, made no regular seasonal movements between separate winter and summer ranges, and displayed little intra- or interannual fidelity to sites previously visited. Nomadic eagles displayed a range of short- and long-distance, irregular movements among multiple locations throughout the year with little interannual consistency in sites visited, seasonal departure dates, or overwintering or summering areas (Fig. 2d).

We classified ten individuals as breeders, five as localized, four as migratory and nine as nomadic (Table 1). Eagles exhibited strongly individualistic movement in terms of travel pathways and locations visited throughout the year, but general movement patterns were consistent within strategies. Migratory and nomadic individuals ranged widely, as far south as Vancouver Island, British Columbia, Canada, and as far north as the Peel River, Yukon Territory, Canada.

**Table 1:** Summary by movement class, sex, and age class

Movement class	Adult		Immature		Total	UD (km^2^)	Dist/day (km)
	♂	♀	♂	♀			
Breeder	5	5	0	0	10	1418 ± 770	8.17 ± 0.44
Localized	2	3	0	0	5	15,418 ± 6895	8.97 ± 0.54
Migratory	3	0	1	0	4	76,897 ± 33,654	21.2 ± 1.68
Nomadic	3	2	0	4	9	39,763 ± 12,219	10.7 ± 0.67

Summary by movement class, sex, and age class of bald eagles monitored along the north Pacific coast, 2010–2016. Areas of utilization distributions (UD) and distance traveled per day are reported ± SE

**Fig. 3:**
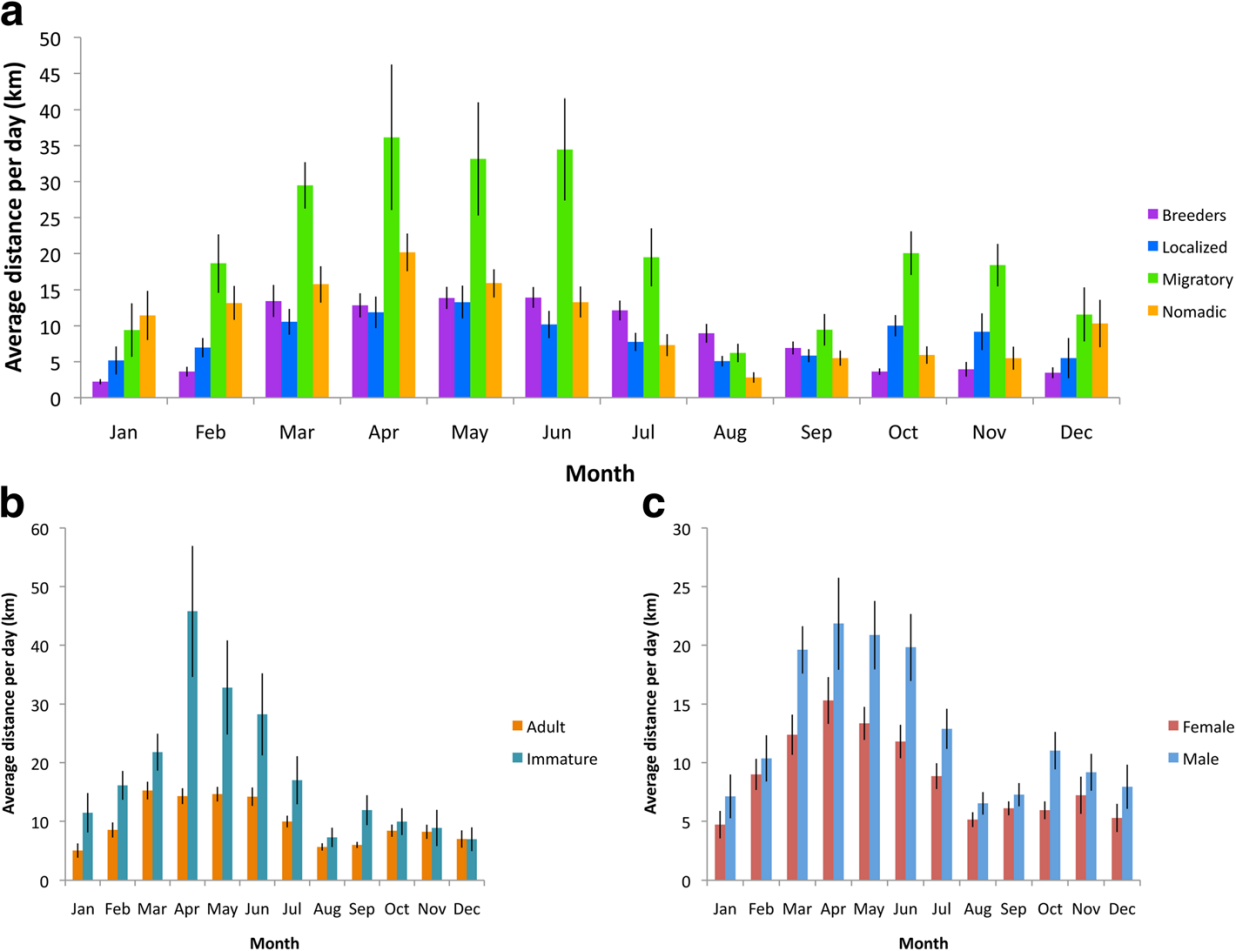
Average distance traveled per day by bald eagles monitored along the north Pacific coast, 2010–2016; data presented by month by **a** movement strategy, **b** age class, and **c** sex class, ± SE. Migratory birds traveled greater distances per day than any other class (*P* < 0.001). Across all months, immature birds traveled greater distances per day than adults (*P* < 0.0001), and males traveled greater distances per day than females (*P* < 0.001)

Across all movement strategies, distances traveled were highest in spring (*P* < 0.0001), at an average of 17.4 ± 1.1 km/day, followed by winter (11.4 ± 0.81 km/day) and autumn (8.1 ± 0.63 km/day), and were lowest in summer months (7.8 ± 0.47 km/day; Fig. 3a). Migratory birds traveled greater daily distances (21.2 ± 1.7 km/day) than any other movement class (*P* < 0.001; Table 1). Nomadic birds traveled significantly farther (10.7 ± 0.67 km/day) than breeders (8.17 ± 0.44 km/day; *P* < 0.05). No significant differences existed in daily movement between breeders and localized birds (8.97 ± 0.54 km/day) or between nomadic birds and localized birds. Migratory eagles also had the largest ranges of any movement strategy (76,897 ± 33,654 km^2^; *P* < 0.0001; Table 1), followed by nomadic eagles (39,763 ± 12,219 km^2^), localized eagles (15,418 ± 6895 km^2^), and breeding eagles (1418 ± 770 km^2^).

Across all movement strategies and across all months, immature birds traveled significantly farther per day (18.9 ± 1.8 km/day) than adults (10.1 ± 3.7 km/day; *P* < 0.0001; Fig. 3b). Male eagles (13.3 ± 0.69 km/day) traveled significantly farther than females (9.1 ± 0.42 km/day; *P* < 0.001; Fig. 3c).

### Space use

**Fig. 4:**
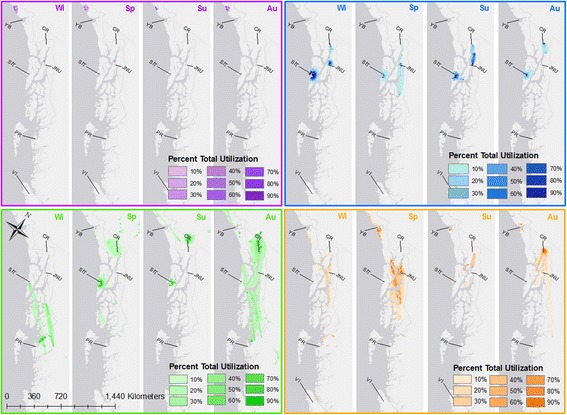
Utilization distribution (5 km^2^ raster cell size) from BBMM of breeders (*purple*), localized (*blue*), migratory (*green*), and nomadic (*orange*) bald eagles monitored along the north Pacific coast, 2010–2016 for each season (Wi = winter, Sp = spring, Su = summer, Au = autumn). For each strategy, the darkest color represents the minimum area to achieve 10% of the total utilization. YB = Yakutat Bay, CR = Chilkat River, SIT = Sitka, JNU = Juneau, PR = Prince Rupert, VI = Vancouver Island

Intensity of use, as revealed through dBBMM varied by season and movement strategy (Fig. 4). Breeders remained on or near nesting sites year-round. Non-breeding localized eagles ranged more widely than breeders, engaging in short-distance travel, particularly in spring, but use was generally restricted to areas of Sitka, Juneau, and the Chilkat River near their respective locations of capture. Migratory and nomadic eagles used the north Pacific coast much more extensively. Migratory individuals shifted habitat use seasonally, with migratory corridors highlighted in autumn (southward migration) and late winter (northward migration). Nomadic individuals had the highest intensity of use across the greatest area, particularly in spring (Fig. 4).

## Discussion

### Movement strategies

Understanding the extent of intraspecific variation in movement strategies can provide important insights into population structure and distribution. We found evidence of four different movement strategies in north Pacific bald eagles, which are likely associated with the age, sex, and breeding status of individuals. Given that anadromous fish and other resources available in coastal systems vary across broad scales but are temporally predictable [15, 36, 37], we anticipated that migratory movement strategies would be favored, but migratory individuals represented the smallest contingent of the eagles we tracked. Much larger proportions of the non-breeding eagles we followed were either localized or nomadic. However, given that our sample size was only 28 individuals, it is difficult to generalize and it is possible that migratory individuals represent a larger proportion of the total population than is captured here, as is suggested by results from coastal British Columbia, Canada [22]. It is also important to note that, while none of our migratory individuals bred, it is possible that some breeding individuals do migrate instead of remaining near a nesting site year-round.

Though sample sizes were small, some patterns did emerge within movement strategies. All breeding and localized individuals were adults. This result confirms that seen by Hodges et al. [20] and Kralovec [21] in southeastern Alaska where adults were residents that made small movements to locally abundant food resources at times but remained on one range year-round. While previous studies only identified a single class (i.e. residents), we were able to differentiate two classes, breeders and non-breeding localized birds, among resident adults.

There was a relatively even distribution of the sex classes among breeders, localized, and nomadic individuals, but all of the migratory individuals we captured were male. Since sample sizes were small, it is likely that some female eagles in the region are migratory. However, bald eagles are sexually dimorphic—females weigh around 25% more than males [18]—and larger eagles are more successful than smaller individuals in contests for food [38]. As such, it is possible that female eagles are better competitors when food becomes limiting, and thus males are more likely to adopt alternative movement strategies in search of food. This is supported by our finding that, across all movement strategies, males traveled farther per day than females throughout the year.

Within movement strategies, breeders generally remained near nesting sites year-round. This relative immobility could indicate a lack of suitable nesting locations relative to the number of available breeders in the population. Raptor populations often contain substantial numbers of non-territorial, non-breeding adults known as floaters [39, 40]. Previous research has identified a high number of non-breeding adult eagles along the north Pacific coast [41]. The existence of these non-breeding floaters implies that breeding opportunities are limited, allowing us to infer that floaters arise in a population because territorial pairs occupy all suitable nesting habitat [40]. Our data support this hypothesis, suggesting that suitable nesting habitat in southeastern Alaska is occupied by breeding bald eagles. Breeding individuals may have to forego access to seasonally abundant resources in distant locations in favor of holding nesting territory outside the breeding season, and supplemental adults may adopt a localized movement strategy to utilize some seasonal resources but remain available to capitalize on openings in the breeding population when available.

Breeders, localized, and migratory eagles all tended to use sites that had been visited before, often many (11 or 12) months prior. This suggests that bald eagles may have a cognitive map that allows them to navigate to previously used locations, as well as a sense of the timing of different anadromous and other fish runs throughout the year. To a much lesser extent, some nomadic individuals revisited sites as well. For example, at least four of the seven nomadic individuals captured on the Chilkat River returned to the area the following autumn to feed on chum salmon (*O. keta*), and one nomadic female revisited the same eulachon (*Thaleichthys pacificus*) run during two successive springs. Nomadism in animals is generally thought to be associated with unpredictable patchiness of resources [10]. Predictability, however, can be defined both by the spatial and temporal consistency of the resource availability and the awareness of the animal of the timing and location of the resource; i.e., a resource that is spatially and temporally predictable may seem unpredictable to an animal with incomplete knowledge of the resource’s availability. It is possible that nomadic individuals do not yet have the requisite knowledge of the landscape and lack the experience necessary to reliably reencounter resource pulses on an annual basis. Four out of the five immature eagles we captured adopted a nomadic movement strategy, and immature eagles moved greater distances than adults throughout the year. As these individuals mature and gain familiarity with the timing and spatial location of resources, their movement strategies might shift toward migratory or localized strategies. While we did not observe any individuals shift their strategy year to year (i.e. a nomadic individual shifting to a migratory strategy, or a migratory animal shifting to a localized strategy), it is possible that eagles will alter their strategy based on age and/or change in breeding status. Future research should examine if and how strategies of movement change within individuals over time.

### Space use

We found that areas of highest-intensity space use varied among different movement strategies (Fig. 4). Space use of breeding eagles was generally limited, suggesting that favored nest sites might be located in areas where heterogeneity in resource availability is relatively low. While localized eagles were not as constrained in their space use as breeders, most of their space use was concentrated around their respective capture locations, with occasional longer-distance forays for access to seasonal resources, primarily in autumn and spring. Across all movement strategies and across both sex and age classes, distance traveled per day was highest in spring, and was most constrained during summer months. Summer months coincide with the peak of the spawning season for most populations of Pacific salmon in southeastern Alaska. We suggest that movement during summer months is lowest across all individuals as a result of salmon availability. Regional salmon availability throughout southeastern Alaska is high, and heterogeneity in the dispersion of salmon and the timing of spawning events extends eagles’ access to salmon across multiple months [36]. Thus, Pacific salmon represent a highly predictable and temporarily widespread food resource for bald eagles.

Research on eagle space use in this system would benefit from modeling linking external variables, such weather patterns, intraspecific competition, fish abundance, and landscape characteristics to eagle movement towards a mechanistic understanding of these varying movement strategies. Accurate measurement of some of these variables, however, poses a serious challenge, especially at a meaningful landscape scale. While many Pacific salmon streams are monitored for salmon abundance throughout southeastern Alaska and British Columbia, data for many runs are often collected once a season or irregularly, as opposed to frequent intervals that more closely parallel the time scales on which eagles feed. Additionally, other anadromous species important for eagles and other wildlife, such as eulachon and Pacific lamprey (*Lampetra tridentata*), are not regularly monitored, and little information exists regarding abundance and spawning timing and duration for these species.

### Implications for management

Conservation strategies for highly mobile species such as eagles have largely focused on migration, operating on the assumption that animals are making regular, annual movements between distinct seasonal ranges. One consequence of this assumption is that conservation efforts have generally targeted protection of habitats associated with migratory movements [42–45]. In migratory or partially migratory bird species, these habitats typically include migratory stopover points, overwintering areas, and specific migration corridors. However, in a population with high intraspecific variation in movement strategies, any management plan tailored only toward migratory individuals risks altering the sex or age class structure of the population. For example, neither protection of migratory corridors or protection of seasonal ranges will sufficiently account for the movement of nomadic individuals. The presence of nomads in the population makes protection of movement corridors largely infeasible, as nomadic individuals are moving irregularly across the landscape at large spatial scales. Further, protected movement corridors would have little benefit to the largely residential breeding individuals, or to localized individuals who engage in primarily short-distance travel. It is possible that a focus on protecting important regional food resources for north Pacific eagles will sufficiently account for intraspecific variation in movement strategies. Identification of critical resources, however, may be a challenging endeavor given the heterogeneity of resource availability in the region.

## Conclusions

Alternative movement strategies among north Pacific eagles are likely associated with the age and sex class, as well as breeding status, of an individual. Intraspecific variation in movement strategies within the population results in different space use patterns among contingents. Effective conservation and management of north Pacific bald eagles will require a framework that recognizes the presence of alternative movement strategies among individuals of different sex and age classes in the population.

## Additional file

Additional file 1: Capture locations, age and sex classes, movement classifications, and tracking periods for 28 bald eagles (*Haliaeetus leucocephalus*) in southeastern Alaska. (DOCX 15 kb)
