# Supplementary material for: To migrate, stay put, or wander? Varied movement strategies in bald eagles (Haliaeetus leucocephalus)
Source: Mov Ecol. 2017 May 5;5:9. doi: 10.1186/s40462-017-0102-4 (PMC5418703; doi:10.1186/s40462-017-0102-4)
Supplement: Additional file 1: — Capture locations, age and sex classes, movement classifications, and tracking periods for 28 bald eagles (Haliaeetus leucocephalus) in southeastern Alaska. (DOCX 15 kb) [file 40462_2017_102_MOESM1_ESM.docx]

**Additional file 1**

| **Individual** | **Sex** | **Age** | **Movement** | **Capture Site** | **Tracking Period** | | **N Loc** |
| --- | --- | --- | --- | --- | --- | --- | --- |
| 49525 | ♀ | Adult | Breeder | Icy Bay | 19 Jul 2010 | 4 Sep 2012 | 5399 |
| 49526 | ♂ | Adult | Breeder | Icy Bay | 22 May 2011 | 3 Jan 2016 | 14433 |
| 495271 | ♀ | Adult | Breeder | Icy Bay | 29 May 2010 | 29 Jun 2011 | 4182 |
| 495272 | ♂ | Adult | Breeder | Icy Bay | 19 May 2012 | 4 Jan 2016 | 10444 |
| 49528 | ♂ | Adult | Breeder | Icy Bay | 22 Jun 2011 | 5 Jun 2012 | 3583 |
| 49532 | ♀ | Immature | Nomadic | Icy Bay | 25 May 2010 | 3 Sep 2011 | 5062 |
| 49545 | ♀ | Adult | Breeder | Icy Bay | 23 Jul 2011 | 5 Nov 2015 | 11557 |
| 49547 | ♂ | Adult | Breeder | Icy Bay | 16 May 2010 | 6 Nov 2012 | 9111 |
| 49553 | ♀ | Adult | Breeder | Icy Bay | 20 Jul 2011 | 18 Nov 2012 | 4524 |
| 103318 | ♀ | Adult | Nomadic | Sitka | 19 Mar 2011 | 16 Oct 2015 | 14323 |
| 103319 | ♀ | Adult | Localized | Sitka | 17 Mar 2011 | 26 Dec 2015 | 15854 |
| 103322 | ♂ | Adult | Localized | Sitka | 18 Mar 2011 | 8 Jul 2014 | 8567 |
| 103326 | ♀ | Adult | Localized | Juneau | 25 Mar 2011 | 17 Jan 2014 | 7140 |
| 103327 | ♀ | Adult | Nomadic | Chilkat River | 4 Nov 2013 | 10 Oct 2015 | 6327 |
| 103328 | ♂ | Adult | Migratory | Sitka | 19 Mar 2011 | 30 Apr 2013 | 5658 |
| 103330 | ♂ | Adult | Migratory | Sitka | 17 Mar 2011 | 8 Nov 2015 | 13581 |
| 107178 | ♂ | Adult | Nomadic | Chilkat River | 5 Nov 2013 | 5 Nov 2015 | 7156 |
| 107179 | ♂ | Adult | Nomadic | Chilkat River | 5 Nov 2013 | 28 Oct 2015 | 6876 |
| 107180 | ♂ | Adult | Localized | Chilkat River | 5 Nov 2013 | 29 May 2014 | 1731 |
| 107181 | ♂ | Adult | Nomadic | Chilkat River | 5 Nov 2013 | 27 Jun 2015 | 5732 |
| 122094 | ♀ | Adult | Localized | Chilkat River | 5 Nov 2012 | 3 May 2015 | 5158 |
| 122095 | ♂ | Adult | Migratory | Chilkat River | 5 Nov 2012 | 1 Nov 2015 | 12638 |
| 122096 | ♂ | Adult | Breeder | Juneau | 9 Jun 2014 | 27 Feb 2015 | 1580 |
| 122519 | ♀ | Immature | Nomadic | Chilkat River | 5 Nov 2012 | 6 Nov 2015 | 11611 |
| 122795 | ♂ | Immature | Migratory | Chilkat River | 4 Nov 2012 | 4 Nov 2015 | 15184 |
| 133480 | ♀ | Immature | Nomadic | Chilkat River | 4 Nov 2013 | 3 Oct 2015 | 4490 |
| 133481 | ♀ | Immature | Nomadic | Chilkat River | 4 Nov 2013 | 17 May 2015 | 3385 |
| 133482 | ♀ | Adult | Breeder | Chilkat River | 5 Nov 2013 | 27 Oct 2015 | 5148 |
